# What’s in your card? The impact of online food delivery apps on depression and eating behaviors

**DOI:** 10.3389/fnut.2025.1664724

**Published:** 2025-11-04

**Authors:** Tugce Ozlu Karahan, Dila Cakmakci, Eylül Kurtoglu, Zeynep Kul, Irem Sevim Kidan, Emre Batuhan Kenger

**Affiliations:** Department of Nutrition and Dietetics, Faculty of Health Sciences, Istanbul Bilgi University, Istanbul, Türkiye

**Keywords:** depression, digital eating habits, eating behavior, online food delivery application, young adults

## Abstract

**Introduction:**

Digitalization, through smartphones and online platforms, has become deeply embedded in daily life, beginning to exert significant effects on eating habits and psychological health. Online food delivery (OFD) applications (app) provide easy access to fast food and processed products, exposing individuals to a constant digital food environment. Examining the relationship of these applications with eating behaviors and conditions such as depression is particularly important in the context of increasing mental health problems among young adults. This study aims to examine the relationship between the frequency of use of OFD apps and user attitudes toward these apps, with depression level and eating behaviors in young adults.

**Methods:**

Participants’ demographic information, frequency of use of OFD apps, and attitudes toward these apps were determined by questionnaire questions; depression status was assessed using the Beck Depression Inventory; and eating behaviors were assessed using the Three-Factor Eating Scale (TFEQ-R21).

**Results:**

A total of 383 young adults aged 18–35 years participated in our study. The group with the lowest frequency of OFD apps use (21.2 ± 0.4) had significantly lower uncontrolled eating scores than the other groups (23.4 ± 0.7 and 23.3 ± 0.6; *p* = 0.005). In addition, a significant decrease in cognitive restraint levels was observed as the frequency of OFD apps use increased (*p* = 0.031). In addition, depression scores of individuals with more OFD apps (4–6) on their phones (14.1 ± 1.3) were found to be higher than those of individuals who did not use any apps (8.8 ± 1.4; *p* = 0.025).

**Discussion:**

The findings of our study suggest that the digital food environment can be a determinant not only of individuals’ physical health but also of their psychological health and behavioral eating habits.

## 1 Introduction

The rapid advancement of the digital age from its inception to the present has significantly transformed human behavior and habitual patterns (1). As the demand for digital communication tools and their role in daily life has increased, online food delivery (OFD) applications (apps) have also become widespread (2). The development and use of food distribution applications worldwide have changed how food suppliers and consumers interact (3). Smartphone apps allow customers to order menu items or nutrients from various food service outlets for pick-up or delivery by freelance couriers (4). These apps have encouraged individuals to meet their shopping needs online, and online food and grocery shopping has increased considerably during the lockdowns initiated in response to the coronavirus disease (COVID-19) pandemic in 2019, as well as the fear of the spread of the virus. (1, 5). According to a survey by the Turkish Statistical Institute, the proportion of consumers in Turkey purchasing goods or services online increased from 44.3% in 2021 to 46.2% in 2022. The study revealed that 50.2% of consumers who purchased goods or services bought OFD services (6).

It has been reported that increased frequency of OFD apps use is associated with changes in individuals’ eating habits, including a higher frequency of snacking, a preference for sweet and ultra-processed foods over fruits, vegetables, and fresh produce, and a decline in adherence to healthy dietary practices (7). Supporting this trend, a study evaluating the nutritional quality of the best-selling OFD set menus in China found that scores ranged from 15 to 85, with an average of 36.57 out of a possible 100. Notably, 89.56% of the menus scored below 50, indicating poor nutritional quality (8). Both the low dietary quality patterns of menus and people’s preference for these products (9) have been associated with increased body mass index (10) and other public health problems (11, 12). A systematic review examining diet-related chronic disease risk factors associated with the use of OFD apps during the pandemic evaluated a total of 53 studies. The results of nine of these studies classified the potential effects of OFDs on health as either positive or negative. Eight of the findings were associated with adverse diet-related chronic disease risk factors, while only one was reported as health-promoting. Reported adverse effects of OFD use included perceived obesity status, weight gain, emotional eating, snacking behaviors, and a tendency toward ordering less healthy foods (7). Furthermore, reports from the most frequently used platforms indicated that among the most commonly ordered foods by American consumers were high-calorie options such as cheeseburgers, French fries, pizzas, nachos, cheesecake, pork ribs, and chicken & waffle sliders. Over the past three decades, increases in fast-food portion sizes, calorie content, and sodium levels have further heightened concerns that these applications may exacerbate the ongoing obesity epidemic (13). Collectively, these findings suggest that adverse health outcomes such as overweight and obesity—well-established risk factors for noncommunicable diseases (NCDs)—may be further reinforced through the use of OFD apps.

Fundamental motivations, such as the ease of use of OFD apps (14)—combined with the widespread promotion, availability, and accessibility of energy-dense foods—may contribute to disordered eating behaviors and trigger cognitive states that promote excessive food consumption (15). These apps use personalized advertising and marketing tactics to draw users’ attention to specific foods, making it difficult to maintain a balanced eating habit, especially for individuals with irregular eating behaviors (16–18). Frequent consumption of food ordered through online platforms has been suggested to be associated with a heightened risk of disordered eating behaviors, including emotional eating and uncontrolled eating (2). Difficulties in regulating negative emotions have been identified as a contributing factor to the exacerbation of eating disorder symptoms (2, 19). In addition, it is emphasized that people’s mood is one of the most important reasons for impulsive buying behavior in online food and grocery shopping (20). Depression has been associated with more irregular eating patterns and has been shown to influence digital dietary behaviors (21). In light of all this information, this study aims to reveal the frequency of use of OFD apps and the relationship between attitudes toward these apps and depression and eating behaviors in young adults within the framework of the habit changes brought by the digital age. In this study, our first hypothesis is that the frequency of using online food delivery (OFD) applications is associated with individuals’ eating behaviors and depression levels. Our second hypothesis is that having a greater number of OFD applications on one’s phone leads to differences in eating behaviors and depression levels. Our third hypothesis is that participants’ attitudes toward OFD applications (such as the influence of coupons, healthy eating motivation, or mood when using the apps) are significantly related to both eating behaviors and depression.

## 2 Methods

### 2.1 Study population

**Figure 1:**
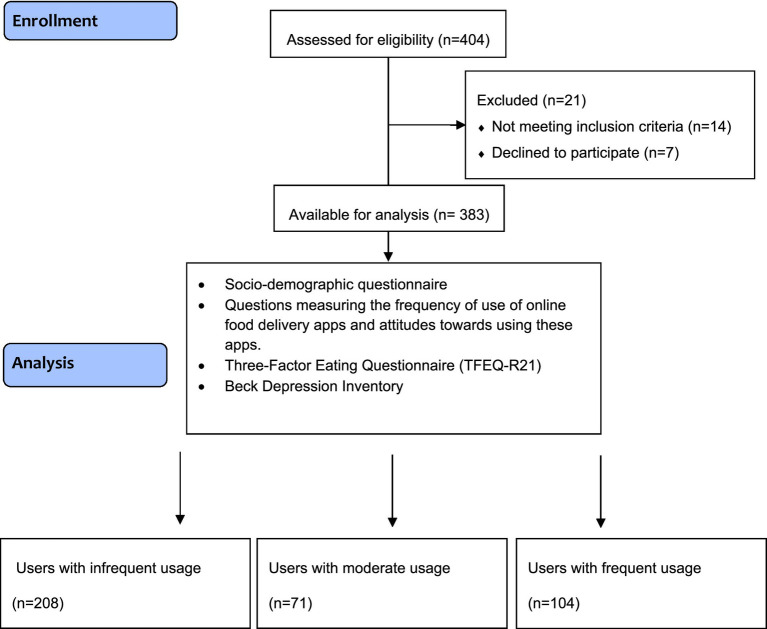
Flow diagram of the study.

This cross-sectional study involved young adults aged 18 to 35 who regularly use OFD apps (22). Exclusion criteria included individuals with a current or past diagnosis of an eating disorder or depression, those under 18 or over 35 years of age, and individuals who did not provide informed consent. The study initially included 404 young adults. However, 8 individuals with a diagnosis of depression, 6 with a diagnosed eating disorder, 14 who did not meet the inclusion criteria, and 7 who declined to participate were excluded from the final sample. The study was completed with a total of 383 individuals. After the completion of the study, the sample size was evaluated using the G*Power 3.1.9.4 software. A *post hoc* power analysis was conducted for the three groups, based on the mean and standard deviation values of the cognitive restraint scores across the three categories of OFD usage frequency (low, medium, high). With a significance level of 5% (*α* = 0.05) and the calculated effect size (*f* = 0.16), the statistical power of the study was determined to be 80.7%. The Consort Flow Chart of the study is shown in Figure 1. Ethical approval of the study was obtained from Istanbul Bilgi University Human Research Ethics Committee, which was prepared by the ethical standards of the Declaration of Helsinki (Approval No: 2024–10,160-204, Date: 21.11.2024). Participant recruitment was conducted between December 2024 and April 2025 through word-of-mouth and announcements made via social media platforms (Facebook, Twitter, Instagram). Data were collected using an online survey administered via Google Forms. At the beginning of the survey, participants were provided with an information sheet outlining the study’s purpose, scope, and the principles of voluntary participation, and it was emphasized that the data would be used solely for scientific purposes. Written informed consent was obtained online from individuals who agreed to participate. This method was chosen particularly because young adults are active users of digital platforms and find completing surveys online more practical. In addition, the use of an online survey allowed for access to a broader sample without the limitations of time and place. Furthermore, participants’ frequency of OFD app use was self-reported and categorized into three groups: infrequent use (three times per month or less), moderate use (once or twice per week), and frequent use (three times per week or more). This classification allowed for the comparison of groups by distinguishing low, moderate, and high levels of usage. In addition, this approach was deemed appropriate for evaluating the potential dose–response relationship between usage frequency and psychological as well as behavioral outcomes.

### 2.2 Usage and attitudes toward online food delivery applications

The questionnaire developed for this study included various items to assess the frequency of OFD apps use and participants’ attitudes toward these apps. Participants were asked to report their usage frequency weekly or monthly. Based on participants’ responses, the frequency of application use was categorized by the researchers into three groups: infrequent use (three times per month or less), moderate use (one to two times per week), and frequent use (three times per week or more). The number of OFD apps installed on participants’ smartphones was also assessed as a separate variable, categorized into three groups: 0, 1–3, and 4–6 applications.

Participants were also asked whether there was any change in the frequency of their use of such apps during the pandemic, whether these apps affected their motivation to eat healthy, the impact of discount coupons offered in the apps on their desire to eat, and the external factors (e.g., social media advertisements, weather, fatigue) that affected their choice to use these apps. In addition, the mood in which participants used these apps (happy, sad, stressed, etc.) was also questioned.

These variables in the questionnaire form were used both in descriptive statistical analyses and in multivariate analyses in relation to eating behaviors and depression scores.

### 2.3 Assessment of depression status

Individuals’ susceptibility to depression was measured with the Beck Depression Inventory. Beck Depression Inventory is a self-assessment scale created by Beck in 1961 to evaluate the specific attitudes and symptoms of depression (23). Hisli conducted the Turkish validity and reliability study of the scale in 1989 (24). In this scale, which consists of 21 self-assessment statements in total, each sentence is scored with four different options. While the options are evaluated in the range of 0–3, total scores can vary between 0 and 63. As the score increases, depression symptoms increase. Cronbach’s Alpha value of the scale was found to be 0.80.

### 2.4 Assessment of eating behavior

Eating behaviors of individuals were determined with the three-factor eating scale. The Three-Factor Eating Scale, which was first developed by Stunkard et al. (25), finalized by Cappelleri et al. (26) as the “Three-Factor Eating Scale (TFEQ-R21)” and adapted into Turkish by Karakuş et al. (27), is used to measure the behavioral and cognitive components of eating. All of the items in the questionnaire are in a 4-point Likert type, and the answers are scored as “absolutely wrong” 1, “mostly wrong” 2, “absolutely right” 3, and “mostly right” 4. The questionnaire shows a 3-factor structure within itself: Uncontrolled eating refers to losing control while eating as a result of hunger or any external stimulus and includes 9 items (items 3, 6, 8, 9, 12, 13, 15, 19, and 20). The minimum score for this sub-factor is 9, and the maximum score is 36. Cognitive restriction refers to consciously restricting food intake to control body shape and weight and includes 6 items (items 1, 5, 11, 17, 18, and 21). The minimum score for this sub-factor is 6, and the maximum score is 24. Emotional eating examines overeating in negative emotional states (anger, sadness, stress, etc.) and includes 6 items (items 2, 4, 7, 10, 14, and 16). The minimum score for this sub-factor is 6, and the maximum score is 24. A higher score in any sub-factor in the questionnaire indicates that the eating behavior related to that factor is more dominant.

### 2.5 Statistical analysis

The data obtained were statistically evaluated in SPSS (Statistical Package for the Social Sciences) 30.0 package program. Statistical significance was accepted as *p* < 0.05 in all analyzes. Number, percentage, mean, standard deviation, standard error, median, 25th, and 75th percentile values were used in descriptive statistics. According to OFD usage frequency, categorical variables including gender, educational status, employment status, presence of chronic disease, and attitudes toward OFD use were evaluated using Chi-square analysis. After checking the conformity of the data to the normal distribution, the t-test or the Mann–Whitney U test was used for normal pairwise group comparisons. For comparisons of more than two groups, one-way ANOVA or Kruskal-Wallis test was used. Adjusted means of eating behavior subscore and total scores and depression scores (age, gender, body mass index, education level, employment status, presence of chronic diseases) were compared using analysis of covariance (ANCOVA) across the number and frequency of use of OFD apps. Before this analysis, depression scores were logarithmically transformed to reduce the effect of outliers and to obtain a more normal distribution suitable for parametric tests. Multiple linear regression analysis was applied to determine the effect of OFD practices and depression on eating behavior sub-scores and total scores. To determine *β* and 95% confidence intervals, models adjusted for age, gender, body mass index, education level, employment status, and presence of chronic diseases were used. In the multiple linear regression analysis, model assumptions were tested. The distribution of residuals, linearity, and homoscedasticity were examined graphically and found to be appropriate. Correlation coefficients among independent variables were below 0.80, indicating no concerns regarding multicollinearity. In addition, Cook’s distance and leverage values were assessed, and no influential observations were detected. The covariates used in the multiple linear regression analysis (age, gender, body mass index, educational level, employment status, and presence of chronic disease) have been identified in the literature as important sociodemographic and health-related variables influencing depression and eating behaviors. For this reason, these variables were included in the model as covariates. Furthermore, the study was completed with 383 participants, and the sample size was found to be adequate based both on the results of the power analysis and on the general rule of “at least 10–15 observations per covariate” recommended for multiple regression (28).

## 3 Results

### 3.1 Results on participants’ general characteristics and OFD usage/attitudes

The final sample consisted of 383 young adults, with a median age of 23.0 years (IQR: 22.0–25.0), of whom 56.7% were female. According to the Kruskal-Wallis test conducted to analyze the general characteristics of participants across OFD frequency categories, age significantly decreased with increasing OFD usage frequency (*p* < 0.001). In contrast, median body weight (67.0 kg, IQR: 56.0–83.0) and BMI (23.2 kg/m^2^, IQR: 20.4–26.2) did not vary significantly across groups (*p* = 0.752 and *p* = 0.994, respectively). Among participants with a bachelor’s degree or higher, 28.6% were frequent users, whereas this rate was 19.0% among those with a high school education or less (*p* = 0.003). The general characteristics of participants based on the frequency of OFD app use are shown in Table 1.

**Table 1:** General characteristics of participants according to frequency of use of food delivery apps.

Parameters	Infrequent (*n* = 208)	Moderate (*n* = 71)	Frequent (*n* = 104)	Total (*n* = 383)	*p*
Age (years)	24.0 (22.0–26.0)	22.0 (21.0–24.0)	23.0 (22.0–24.0)	23.0 (22.0–25.0)	**<0.001**
Gender					0.265
Male	84 (50.6)	30 (18.1)	52 (31.3)	166 (43.3)	
Female	124 (57.1)	41 (18.9)	52 (24.0)	217 (56.7)	
Body weight (kg)	66.0 (56.2–82.0)	68.0 (56.0–82.0)	68.0 (57.0–84.7)	67.0 (56.0–83.0)	0.752
Body mass index (kg/m^2^)	23.3 (20.5–26.2)	22.9 (20.5–26.7)	23.3 (20.3–26.5)	23.2 (20.4–26.2)	0.994
Educational status					**0.003**
High school and below	43 (74.1)	4 (6.9)	11 (19.0)	58 (15.1)	
Bachelor’s degree and above	165 (50.8)	67 (20.6)	93 (28.6)	325 (84.9)	
Employment status					0.067
Employed	110 (60.4)	28 (15.4)	44 (24.2)	182 (47.5)	
Unemployed	98 (48.8)	43 (21.4)	60 (29.9)	201 (52.5)	
Presence of chronic disease					0.525
Yes	28 (62.2)	7 (15.6)	10 (22.2)	45 (11.7)	
No	180 (53.3)	64 (18.9)	94 (27.8)	338 (88.3)	
Changes in the use of online food delivery apps during the pandemic					0.203
Increased	86 (50.3)	38 (22.2)	47 (27.5)	171 (44.6)	
Remained	122 (57.5)	33 (15.6)	57 (26.9)	212 (55.4)	
The effect of coupons defined in online food delivery apps on the desire to eat					**0.038**
Yes	115 (49.6)	51 (22.0)	66 (28.4)	232 (60.6)	
No	93 (61.6)	20 (13.2)	38 (25.2)	151 (39.4)	
The impact of online food delivery apps on healthy eating motivation					**0.045**
Yes	26 (40.6)	17 (26.6)	21 (32.8)	64 (16.7)	
No	182 (57.1)	54 (16.9)	83 (26.0)	319 (83.3)	
Reasons for choosing online food delivery apps?					0.064
Time saving	60 (60.6)	18 (18.2)	21 (21.2)	99 (25.8)	
Easy accessibility	137 (50.6)	52 (19.6)	81 (29.9)	270 (70.8)	
Other	11 (78.5)	1 (7.2)	2 (14.3)	14 (3.4)	
Mood when choosing online food delivery apps					**0.028**
Sad	32 (55.2)	14 (24.1)	12 (20.7)	58 (15.1)	
Happy	76 (49.0)	39 (25.2)	40 (25.8)	155 (40.5)	
Stressed	44 (62.0)	7 (9.9)	20 (28.2)	71 (18.5)	
Other	56 (56.6)	11 (11.1)	32 (32.3)	99 (25.8)	
External factors influencing the use of online food delivery apps					0.111
Social media ads	37 (60.7)	12 (19.7)	12 (19.7)	61 (15.9)	
Weather	20 (74.1)	2 (7.4)	5 (18.5)	27 (7.0)	
Fatigue	13 (53.2)	47 (19.0)	69 (27.8)	248 (64.8)	
Other	19 (40.4)	10 (12.3)	18 (38.3)	47 (12.3)	

Chi-square analysis was performed for categorical variables, and Kruskal Wallis analysis was performed for continuous variables. Values are expressed as median (25th-75th percentile) or number (%). Statistical significance (*p* < 0.05) is indicated in bold.

### 3.2 Effects of OFD usage frequency and number of apps on eating behaviors and depression

Table 2 presents participants’ eating behavior and depression scores by both the frequency of OFD use and the number of apps. Uncontrolled eating scores were significantly lower among infrequent users (21.2 ± 0.4) compared to moderate (23.4 ± 0.7) and frequent users (23.3 ± 0.6; *p* = 0.005). Cognitive restraint was lowest in the frequent-use group (12.7 ± 0.4) compared to infrequent users (14.1 ± 0.3; *p* = 0.031). Regarding the number of apps, participants with no apps reported lower depression levels (8.8 ± 1.4) than those with 1–3 apps (10.8 ± 0.4) or 4–6 apps (14.1 ± 1.3; *p* = 0.025).

**Table 2:** Eating behaviors and depression scores of participants according to the number and frequency of use of online food delivery apps.

Scale scores	Frequency of use of online food delivery apps				Number of online food delivery apps			
	Infrequent (*n* = 208)	Moderate (*n* = 71)	Frequent (*n* = 104)	*p*	None (*n* = 39)	1 and 3 (*n* = 305)	4 and 6 (*n* = 39)	*p*
Uncontrolled eating score	21.2 ± 0.4	23.4 ± 0.7	23.3 ± 0.6	**0.005**	21.1 ± 1.0	22.2 ± 0.3	22.9 ± 1.0	0.473
Cognitive restraint score	14.1 ± 0.3	14.0 ± 0.5	12.7 ± 0.4	**0.031**	14.2 ± 0.7	13.7 ± 0.2	13.1 ± 0.7	0.599
Emotional eating score	12.9 ± 0.4	13.6 ± 0.6	13.1 ± 0.5	0.634	12.7 ± 0.8	13.1 ± 0.3	13.5 ± 0.8	0.834
Total score	48.2 ± 0.8	51.1 ± 1.5	49.1 ± 1.2	0.306	48.1 ± 2.1	49.1 ± 0.7	49.5 ± 2.0	0.886
Depression score	11.1 ± 0.6	10.6 ± 1.0	10.8 ± 0.8	0.671	8.8 ± 1.4	10.8 ± 0.4	14.1 ± 1.3	**0.025**

Values are expressed as mean ± standard error. ANCOVA analysis was performed to estimate adjusted means according to age, gender, body mass index, educational status, employment status, and presence of chronic disease. Statistical significance (*p* < 0.05) is indicated in bold.

### 3.3 Relationship between participants’ attitudes toward OFD and eating behaviors and depression

Table 3 presents eating behavior and depression scores according to participants’ attitudes toward OFD apps. Participants whose use increased during the pandemic reported significantly higher subscale and total eating behavior scores (*p* < 0.05). Those who indicated that coupons influenced their food cravings showed higher scores for uncontrolled eating (*p* = 0.050), emotional eating (*p* = 0.025), and total eating behavior (*p* = 0.050). Interestingly, participants who stated that these apps motivated them to eat healthily also scored significantly higher across all eating behavior subscales and the total score (*p* < 0.05). Participants citing social media advertisements as the main factor for use had higher cognitive restraint scores (15.3 ± 4.9) compared to those influenced by fatigue (13.4 ± 4.6) or other factors (12.3 ± 3.8; *p* < 0.010).

**Table 3:** Eating behaviors and depression scores of participants according to their attitudes toward online food delivery apps.

Situtations	Responses	Uncontrolled eating score	Cognitive restraint score	Emotional eating score	Total score	Depression score
Changes in the use of online food delivery apps during the pandemic	Increased	23.7 ± 6.5	14.4 ± 4.8	14.3 ± 5.6	52.4 ± 13.7	10.0 (5.0–16.0)
	Remained	20.9 ± 6.1	13.2 ± 4.5	12.1 ± 4.9	46.3 ± 11.7	9.0 (4.0–15.0)
	*p*	**<0.001** ^**α**^	**0.009** ^**α**^	**<0.001** ^**α**^	**<0.001** ^**α**^	0.323^β^
The effect of coupons defined in online food delivery apps on the desire to eat	Yes	22.6 ± 6.2	13.8 ± 4.7	13.5 ± 5.3	49.9 ± 12.7	9.5 (5.0–15.7)
	No	21.5 ± 6.6	13.7 ± 4.8	12.4 ± 5.2	47.7 ± 13.3	9.0 (4.0–15.0)
	*p*	**0.05** ^**α**^	0.476^**α**^	**0.025** ^**α**^	**0.05** ^***α***^	0.406^β^
The impact of online food delivery apps on healthy eating motivation	Yes	24.1 ± 6.9	16.5 ± 5.3	14.8 ± 5.8	55.4 ± 16.1	7.0 (3.0–13.8)
	No	21.7 ± 6.2	13.1 ± 4.4	12.7 ± 5.1	47.7 ± 11.9	10.0 (5.0–15.0)
	*p*	**0.004** ^**α**^	**<0.001** ^**α**^	**0.004** ^**α**^	**<0.001** ^**α**^	**0.05** ^β^
Mood when choosing online food delivery apps	Sad	23.6 ± 7.2	14.0 ± 4.8	15.5 ± 5.8	53.2 ± 14.7	12.0 (6.0–18.0)
	Happy	21.7 ± 6.5	13.4 ± 4.8	12.0 ± 4.9	47.2 ± 12.8	9.0 (4.0–13.0)
	Stressed	23.5 ± 5.7	14.9 ± 4.6	15.4 ± 5.0	53.9 ± 11.9	12.0 (7.0–18.0)
	Other	21.1 ± 6.0	13.1 ± 4.5	11.8 ± 4.8	45.9 ± 11.6	9.0 (4.0–14.0)
	*p*	**0.018** ^**†**^	0.068^**†**^	**<0.001** ^**†**^	**<0.001** ^**†**^	**0.005** ^**γ**^
External factors influencing the use of online food delivery apps	Social media ads	22.5 ± 7.1	15.3 ± 4.9	14.1 ± 5.8	52.0 ± 14.8	7.0 (3.5–13.0)
	Weather	21.5 ± 7.8	15.6 ± 5.6	12.2 ± 5.4	49.4 ± 16.3	6.0 (4.0–10.0)
	Fatigue	22.1 ± 6.1	13.4 ± 4.6	13.1 ± 5.1	48.6 ± 12.4	10.0 (5.0–17.0)
	Other	22.2 ± 6.1	12.3 ± 3.8	12.4 ± 5.4	46.9 ± 10.9	11.0 (7.0–14.0)
	*p*	0.915^**†**^	**<0.001** ^**†**^	0.285^**†**^	0.198^**†**^	**0.006** ^**γ**^

α, Independent groups t-test; β, Mann Whitney U test; †, One-way ANOVA test, γ, Kruskal Wallis test were applied. Values are expressed as median (25th-75th percentile) or mean ± standard deviation. Statistical significance (*p* < 0.05) is indicated in bold.

### 3.4 Results from multivariate regression analysis

Multivariate linear regression models assessing the impact of the number and frequency of OFD app use on eating behavior subscale and total scores are presented in Table 4. For uncontrolled eating, both moderate (*β*: 2.296; 95%CI: 0.543, 4.051) and frequent (*β*: 2.177; 95%CI: 0.645, 3.708) application use, as well as depression (*β*: 2.941; 95%CI: 1.194, 4.689), were found to have a significant positive effect (*p* < 0.05). In the case of cognitive restraint, only frequent use showed a significant negative association (*β*: −1.441; 95%CI: −2.598, −0.285; p: 0.015). For emotional eating (*β*: 1.614; 95%CI: 0.106, 3.122; *p*: 0.036) and total eating behavior score (*β*: 4.109; 95%CI: 0.489, 7.729; *p*: 0.026), depression was the sole variable with a significant positive effect.

**Table 4:** Multiple linear regression analysis of eating behavior scores.

Variables	Variables type	Uncontrolled eating score				Cognitive restraint score				Emotional eating score				Total score			
		*β*	95% CI		*p*	*β*	95% CI		*p*	*β*	95% CI		*p*	*β*	95% CI		*p*
			Lower	Upper			Lower	Upper			Lower	Upper			Lower	Upper	
Frequency of use of online food delivery apps	Infrequent (Reference)																
	Moderate	2.296	0.543	4.051	**0.011**	−0.146	−1.469	1.178	0.829	0.738	−0.754	2.229	0.332	2.888	−0.747	6.524	0.119
	Frequent	2.177	0.645	3.708	**0.005**	−1.441	−2.598	−0.285	**0.015**	0.181	−1.123	1.483	0.786	0.916	−2.261	4.091	0.571
Number of online food delivery apps	None (Reference)																
	1 and 3	−0.013	−2.183	2.157	0.991	−0.026	−1.665	1.613	0.975	0.071	−1.777	1.916	0.941	0.031	−4.469	4.531	0.989
	4 and 6	−0.117	−3.085	2.851	0.938	−0.139	−2.381	2.101	0.903	0.297	−2.228	2.821	0.817	0.041	−6.113	6.192	0.991
Depression score	Continuous	2.941	1.194	4.689	**0.001**	−0.446	−1.174	0.882	0.509	1.614	0.106	3.122	**0.036**	4.109	0.489	7.729	**0.026**

Multiple linear regression analysis was performed. Statistical significance (*p* < 0.05) is indicated in bold. Adjustments were made for age, gender, body mass index, educational level, employment status, and chronic disease status. Eating behavior scores (Uncontrolled eating, cognitive restraint, emotional eating and total score) are dependent variables; frequency of use of online food delivery apps, number of online food delivery apps and depression score are independent variables.

## 4 Discussion

This study examined the relationships between the frequency of use of OFD apps and user attitudes, depression level, and eating behaviors in young adults within the framework of habit changes brought by the digital age. The main finding obtained from our study is that as the frequency of use of these applications increases, especially uncontrolled eating behaviors increase, and cognitive restraint levels decrease. In addition, a significant increase in depression scores was also observed with the increase in the number of apps.

The pandemic has emerged as an important factor influencing the use of online food delivery applications. Among the participants, 44.6% reported an increase in their use of OFD applications during the pandemic, while 55.4% indicated no change. These self-reported changes did not significantly differ according to the participants’ current frequency of application use. However, existing literature consistently indicates that online food and grocery shopping increased markedly during the pandemic and that this upward trend persisted in the post-pandemic period (29). This discrepancy may be attributed to the characteristics of the study population, as the research was conducted among young adults, it is possible that their frequency of application use was less influenced by the pandemic, leading to the absence of significant differences.

Coupons, healthy eating motivation, and emotional states have been identified as important factors influencing the use of OFD applications. In the study, frequent users of the apps reported that coupons had an effect on food cravings. Similar to our research, a study examining regular users of OFD apps found that coupon users tended to use the application more frequently, and these users exhibited a mechanism that triggered shopping/delivery (food) behaviors more (30). In the present study, participants who reported that OFD apps increased their motivation to eat healthily were likelier to use these apps than those who did not. Supporting this finding, a separate study demonstrated that after users were given the option to shop online, the proportion of spending on healthy products in their shopping baskets increased by 10.2%. Moreover, healthy product spending rose by 21.7% on a calorie-adjusted basis, and overall nutrient density improved by 10.1%. These results suggest that health-conscious individuals are more likely to make healthier food choices when utilizing online platforms (31). In our study, most participants (40.5%) reported using OFD apps when they felt happy. Similarly, previous research has shown that individuals experiencing positive emotions, such as happiness, are more likely to engage in and derive enjoyment from online shopping experiences (32). In addition fatigue was identified as the most common extrinsic factor influencing the use of OFD apps, reported by 64.8% of participants. Similarly, previous research has highlighted that online and offline food ordering services are often preferred to save time and manage fatigue (33).

Our findings indicate that frequent use of OFD apps is associated with less healthy eating behaviors, particularly increased uncontrolled eating and reduced cognitive restraint. These findings are in line with previous literature indicating that increased use of OFD apps predicts the urge to overeat (2, 19). Similar to our study, in a study conducted on 1,450 university students in China, emotional overeating behavior was significantly higher in participants who consumed these foods 4 or more days a week compared to those who consumed online takeaway food less than 1 day a week. (19). Portingale et al. (n = 483) found that daily experiences of loneliness and negative mood, along with the use of OFD apps, were associated with an increased risk of eating disorders. These findings suggest that such OFD apps may contribute to disordered eating behaviors by providing an easily accessible, food-rich environment in which highly palatable and hedonic food choices are prominently promoted (2). In another study involving 2,583 workers and 898 students, emotional overeating and the use of OFD apps were examined. While 75% of the sample exhibited emotional overeating behavior above the clinical threshold, the use of these applications did not have a significant effect on this behavior. The findings suggest that OFD apps primarily serve as a tool for food access, with their impact shaped by individual characteristics, rather than acting as a direct risk factor for emotional overeating (34). Our findings revealed that individuals who used OFD apps more frequently showed lower levels of cognitive restraint. This result is consistent with the structural nature of dietary restriction psychopathology. Constant exposure to food choices, especially the easy availability of high-calorie and attractive foods, may undermine individuals’ efforts to mentally limit food intake. OFD apps are filled with visual and linguistic stimuli that force the user to make decisions, which may strain attention and impulse control, reducing the capacity to maintain cognitive restraint. As noted by Espel-Huynh et al., the brain’s reward system becomes increasingly sensitized during periods of dietary restraint, heightening the risk of overeating when individuals are exposed to environmental food cues (35). In this context, our findings similarly suggest that maintaining a consistent state of self-control is particularly challenging in environments characterized by high accessibility and abundant food choices. Over time, the cognitive effort required to sustain restraint may diminish, increasing vulnerability to dysregulated eating behaviors.

One of the interesting findings of our study is that individuals who reported that OFD apps increased their motivation to eat healthy also had higher uncontrolled and emotional eating scores. This suggests that despite the desire to eat healthy, some individuals continue to have difficulty controlling their eating behaviors and emotional eating tendencies. Although a study (36) on healthy motivations revealed that OFD apps in Jordan are generally perceived as unhealthy, these apps offer an important opportunity to promote healthy eating. The eating behaviors of individuals with healthy eating motivation have a more complex structure and show differences in both eating control and emotional eating dimensions. In addition to all these findings, the lack of studies showing the effects of OFD apps on eating behaviors indicates the need for further research in this area. In particular, future research should investigate whether the use of OFD apps has different effects on eating behavior in various socio-economic contexts and culinary cultures.

Although the potential link between the use of OFD applications and depression has not been sufficiently investigated in the literature, our findings suggest that having a greater number of food delivery applications may be associated with higher levels of depression. In a cohort study conducted in Australia, it was shown that individuals who were more likely to use takeaway services at age 14, in addition to red meat, refined foods and desserts, were associated with more severe depressive symptoms at age 17. However, this study did not specifically focus on takeaway behaviors, considering them part of Western eating habits (37). In a recent study of 6,417 university students in China, takeaway frequency was significantly associated with depressive symptoms (38). These studies emphasize the negative relationship between the frequency of takeout and emotional well-being and highlight the need to focus on the emotional health of consumers who frequently order takeout. In addition, in our study, depression scores were found to have positive and significant effects on levels of uncontrolled eating and emotional eating. All these data suggest that depression and related uncontrolled or emotional eating behaviors may be common in individuals who frequently order takeout. Therefore, food ordering habits through digital platforms should be examined in more detail in terms of their possible effects on individuals’ eating behaviors and psychological health.

Wearable technologies, which are a new field of study, can also be used in the regulation of OFD and eating behaviors. Recent protocols and empirical studies demonstrate the feasibility of integrating wearable-derived data with machine learning approaches to improve prediction and detection of eating disorder behaviors. Presseller and colleagues describe how psychophysiological sensors can augment ecological momentary assessment (EMA) by detecting negative affective arousal in real time, potentially identifying risk states for binge eating episodes before individuals are consciously aware of them (39). Similarly, another study found that the use of wearable-based physiological data (heart rate, electrodermal activity, environmental skin temperature) with machine learning models achieved high accuracy in detecting eating disorder behaviors in daily life (40). Building on these findings, wearable devices may serve as tools for delivering timely alerts or coping strategies when physiological indicators signal an elevated risk of disordered eating. Furthermore, applying wearable technology to OFD could provide a powerful mechanism for personalizing digital health interventions.

One of the most important strengths of this study is that it examined the relationships between the frequency of use of OFD apps and both depression and eating behaviors in young adults using holistic and multivariate analyses. Although there are several studies in the literature on the relationship of such apps with food quality, obesity, and other metabolic diseases, research focusing on the simultaneous effects of these apps on psychological health (especially depression) and sub-dimensions of eating behaviors (uncontrolled eating, emotional eating, cognitive restraint) is quite limited. In this respect, our study makes an important contribution to the literature as one of the rare studies that comprehensively evaluates the potential effects of the digital food environment on both mental health and eating behaviors. Despite the important findings of our study, it has several limitations. First, the cross-sectional design of the study does not allow us to establish a causal relationship. Secondly, since the study sample consisted only of young adults between the ages of 18–35, the generalizability of the findings to older age groups is limited. In addition, the fact that the data were collected through self-report may include possible information errors that may lead to problems such as recall bias in the participants’ responses.

## 5 Conclusion

This study revealed that the number and frequency of use of OFD apps are significantly related to depression levels and eating behaviors in young adults. In particular, as the frequency of OFD apps use increased, an increase in uncontrolled eating behaviors and a decrease in cognitive restraint levels were observed. These findings suggest that OFD apps are not merely shopping tools but are deeply intertwined with individuals’ mood and eating psychology.

In this regard, it is recommended that future studies compare app content, user experience, and different age groups, and use longitudinal designs to determine causal relationships. When developing public health policies for the digital food environment, it is also important to consider the psychological and behavioral effects of these apps.

## Data Availability

The raw data supporting the conclusions of this article will be made available by the authors, without undue reservation.
